# “It’s not just about you”: International students’ vulnerabilities and capacities during the first phase of the COVID-19 pandemic in Canada

**DOI:** 10.1371/journal.pone.0311514

**Published:** 2024-10-03

**Authors:** Ayisha Khalid, Jessica Naidu, Tanvir C. Turin

**Affiliations:** 1 Department of Family Medicine, Cumming School of Medicine, University of Calgary, Calgary, Alberta, Canada; 2 Department of Community Health Sciences, Cumming School of Medicine, University of Calgary, Calgary, Alberta, Canada; De Montfort University, UNITED KINGDOM OF GREAT BRITAIN AND NORTHERN IRELAND

## Abstract

In Canada, the COVID-19 pandemic was initially characterized by emergency government responses that disrupted daily life, especially for marginalized groups. This study explored the vulnerabilities and capacities of international students studying at a university in Calgary, Canada during the first phase of the pandemic. Guided by the Capacities and Vulnerabilities Analysis framework, we thematically analyzed 11 semi-structured interviews with international students. We found that material vulnerabilities included *balancing finances*, *housing conditions*, *lack of information*, *food inaccessibility*, *reliance on public transport*, and *poor mental health*, social vulnerabilities included *lack of social support*, *culture shock*, and *racism*, and attitudinal vulnerabilities included *“nowhere to go”*, *feeling like a burden*, and *perception of Canada as safe*. Material capacities included *financial support*, *knowledge about pandemic*, and *mental health supports*, social capacities included *local social support* and *multilingualism*, and attitudinal capacities included *resilience*, *religious and spiritual beliefs*, *“it’s not just about you”*, and *reflexivity*. We found overlapping and complex relationships between vulnerabilities and capacities, indicating that while international students’ vulnerabilities were exacerbated and introduced challenges during the pandemic, students uniquely leveraged their capacities to offset and recover from challenges. Findings from this study may be informative for stakeholders involved in disaster responses, especially universities and governments, to support international students’ capacities and address their vulnerabilities.

## 1. Introduction

In Canada, international students are migrants with non-permanent resident status who study at designated learning institutions under a study permit [1]. Attracted by the country’s multiculturalism, quality of education, career opportunities, and pathways to permanent residency [2], the international student population has nearly tripled in the last decade [3]. A total of 235,422 international students were enrolled in Canadian universities in 2019–2020, representing 16% of all enrollments [4]. The federal government’s 2019–2024 International Education Strategy, which allocated $147.9 million to diversifying international student recruitment, is expected to further increase the international student population in Canada [2].

Canada’s international students originate from approximately 225 countries, with over half from China and India [5]. The province of Alberta is among the top four destinations for international students [6], and Calgary is the province’s most populous city [7]. The University of Calgary welcomes international students from over 160 countries [8]. Although a diverse migrant population, research highlights that international students often have similar vulnerabilities, such as financial difficulties, limited social networks, and linguistic barriers [9]. Vulnerabilities are the long-term factors that exist in daily life and can weaken people’s ability to cope with and recover from disaster [10]. Research highlights that international students often have similar capacities too, such as resilience and a constructive sense of self [11, 12]. Capacities are the long-term factors that exist in daily life and can strengthen people’s ability to cope with and recover from disaster [10].

Disasters, such as pandemics, highlight vulnerabilities and therefore present an opportunity to address them [10]. The first phase of the coronavirus disease (COVID-19) pandemic in Canada, classified as the period between January 2020 and June 2020 [13], contributed 8,556 deaths [14]. As an unprecedented and overwhelming situation, the first phase incited an emergency disaster response from the Canadian government [15]. Quarantine protocols, mask mandates, social gathering limits, travel restrictions, and institutional closures were all implemented in an effort to slow the rapid spread of COVID-19 [13]. Emergency support programs, such as the Canadian Emergency Student Benefit (CESB) and Canada Emergency Response Fund (CERB), were also established to cushion the social and economic burdens of the pandemic [16].

Most of the established literature on international students’ vulnerabilities and capacities has focused on natural disasters [17–20]. During natural disasters, linguistic, informational, social, and academic difficulties can impede international students’ ability to cope and recover [9, 19]. Established literature scarcely examines international students’ capacities during disasters. This scarcity of literature may be because most discourse on international students stems from a “deficit model”, which refers to the perception that acculturation in the host country is the primary solution to alleviating international students’ vulnerabilities, rather than leveraging students’ pre-existing strengths as capacities for alleviating their vulnerabilities [11].

Ethnic minorities, migrants, and low socioeconomic populations, among others, were identified as marginalized groups disproportionately vulnerable to the effects of COVID-19 early in the pandemic [21]. Missing from the list of marginalized groups were international students, even though their vulnerabilities can intersect the vulnerabilities of these groups, and, unlike most groups, international students were exempted from many government supports due to their non-permanent resident status [15]. At the same time, international students may have capacities which could have strengthened their ability to cope with the pandemic [22].

To our knowledge, there is a gap in exploring both international students’ vulnerabilities and capacities during pandemics in Canada. Left unexamined, the vulnerabilities of international students may be exacerbated and contribute to future disasters. The purpose of this study was to explore the potential vulnerabilities and capacities of international students studying at a university in Calgary, Canada during the first phase of the COVID-19 pandemic.

## 2. Methods

### 2.1 Study design

We employed a qualitative methodology involving 11 semi-structured interviews with international students conducted between October 2020 and January 2021.

### 2.2 Conceptual framework

The Capacities and Vulnerabilities Analysis (CVA) framework is an analytical tool for disaster preparedness and relief [10]. The CVA framework helps map people’s complex vulnerabilities and capacities during disaster in an organized fashion. The framework is based on the idea that implementing disaster responses with developmental and sustainable impacts first requires identifying people’s vulnerabilities and capacities, because capacities must be leveraged, and vulnerabilities must be reduced, for effective development [10].

In this study, we used the CVA framework’s categorization of vulnerabilities and capacities to capture international students’ complex experiences during the pandemic. Anderson (1989) described the three categories of vulnerabilities and capacities in the CVA framework as material, social, and attitudinal. The material category considers productive resources, skills, and hazards (health, food, housing, information, etc.), the social category considers relations and organization of people (language, family, community, etc.), and the attitudinal category considers internal and external perceptions (personal, cultural, religious values, norms, etc.). The three categories of vulnerabilities and capacities may be disaggregated by relevant dimensions, such as gender, age, ethnicity, economic class, and political affiliation as appropriate to further reflect the complexity of reality.

### 2.3 Study participants

For inclusion, participants had to be an undergraduate international student enrolled at the University of Calgary since or before the Winter 2020 semester (since or before January 13, 2020). This was the academic semester disrupted by the first phase of the COVID-19 pandemic. Participants were also to be physically present in Calgary for at least six months following March 13, 2020, which was when the University shifted to online learning due to the pandemic.

Only undergraduate students were included as they tend to vary less in factors like age, financial situation, and marital status than graduate students [23]. Participants were contained to the University of Calgary and Calgary contexts as a step to ensure they had access to similar external supports and structures. If participants left Calgary six or more months after the University’s switch to online learning, they were asked to focus on their experience while physically present in Calgary for the interview.

Participants were recruited using convenience sampling between October 13, 2020 and November 17, 2020. Online advertisements were sent to social media pages of different University ethnic/cultural clubs, emailed to the University’s international student services, and posted to popular informal student networks. Snowball sampling supplemented recruitment. Recruitment continued until code saturation was achieved.

### 2.4 Data collection

Semi-structured interviews helped organize the discussion and enabled participants to provide in-depth information about their experiences [24]. The interview guide (S1 File) was informed by the CVA framework [10], existing disaster-related literature [17–19], consultation with the research team, and pilot work with one international student. The interview guide consisted of a demographic section and an open-ended section. The latter consisted of questions organized by the CVA framework’s categories of vulnerabilities and capacities (material, social, and attitudinal) with inquiries about relevant dimensions, such as gender and ethnicity.

Interviews were conducted using the institutional Zoom platform. Each Zoom meeting required a unique password. Participants were provided an informed consent document at least 24 hours prior to the interview to allow adequate time for reviewing study information. Interviews were audio-recorded and ranged from 30 minutes to 1 hour. Verbal consent, as approved by the ethics board, was witnessed by researchers present at the time of the interview and documented as an audio recording at the beginning of each interview. Interviews were later transcribed verbatim in Microsoft Word. Participant names and any personal identifiers were removed from the data. Data were stored locally, and password protected.

### 2.5 Data analysis

As this study was driven by the CVA framework, we analyzed our data deductively using a recursive process of thematic analysis [25]. We used NVivo 12 to facilitate data organization. We collected and analyzed data simultaneously to monitor the depth and clarity of the narratives. We also used an iterative process to ensure we achieved code saturation in our data collection [26]. We reached code saturation by 11 interviews.

Established notions of trustworthiness were used to increase the rigour of this study [27]. A personal reflective research journal was kept as an audit trail of notable decisions, to facilitate self-reflection, and account for personal values and beliefs throughout the research process. As a way of member checking, we returned the synthesized data to participants with the opportunity to change or remove any parts they believed did not reflect their experience. The participants also had a chance to add aspects that, in their opinion, where missing. All participants provided feedback which was integrated into the synthesis. Ethics approval for this study was granted by the University of Calgary Conjoint Health Research Ethics Board–Ethics ID: REB20-1477.

## 3. Results

Participant demographics are presented in Table 1. Over half of the 11 participants were between 18–20 years of age (63.6%) and female (63.6%). Half were unemployed (54.5%) and almost half were in their second year of undergraduate study (45.5%). Nearly all participants lived with roommates at the time the University switched to online learning, with a majority living on campus (54.5%). Almost all participants had lived in Canada for more than 1 year at the time of the interview (90.9%). Besides English, Urdu was the most common spoken language (27.3%). Most participants were South Asian (36.4%), and the most common countries of origin were Pakistan (18.2%), Nigeria (18.2%), and China (18.2%).

**Table 1:** Participant demographics represented as *n* (%).

Characteristics	Participants (*n* = 11)
**Age**	
Mean (range, standard deviation)	20 years (18–23, 1.5)
18–20 years	7 (63.6)
21–23 years	4 (36.4)
**Current year of undergraduate study**	
Year 2	5 (45.5)
Year 3	2 (18.2)
Year 4	4 (36.4)
**Gender**	
Female	7 (63.6)
Male	4 (36.4)
**Employment status**	
Employed part time	5 (45.5)
Unemployed	6 (54.5)
**Languages spoken fluently**	
Arabic	1 (9.1)
Bengali	1 (9.1)
Chinese	2 (18.2)
English	11 (100)
French	1 (9.1)
Punjabi	1 (9.1)
Spanish	2 (18.2)
Urdu	3 (27.3)
Yoruba	1 (9.1)
**Ethnicity**	
African	2 (18.2)
East Asian	2 (18.2)
Latinx	2 (18.2)
Middle Eastern	1 (9.1)
Multiethnic	1 (9.1)
South Asian	4 (36.4)
**Country of origin**	
Bangladesh	1 (9.1)
China	2 (18.2)
Colombia	1 (9.1)
Egypt	1 (9.1)
India	1 (9.1)
Nigeria	2 (18.2)
Pakistan	2 (18.2)
Peru	1 (9.1)
**Length of stay in Canada, when interviewed**	
1 year	1 (9.1)
More than 1 year	10 (90.9)
**Type of residence in Calgary, in March 2020**	
Off-campus with roommates	4 (36.4)
On-campus individually	1 (9.1)
On-campus with roommates	6 (54.5)

Using the CVA framework’s categories of vulnerabilities and capacities, our analysis yielded six themes and 21 sub-themes (Table 2), which are presented below using illustrative participant quotations.

**Table 2:** Themes and sub-themes identified from interviews using the Capacities and Vulnerabilities Analysis framework.

Themes		Sub-themes
**Vulnerabilities**	**Material**	• Balancing finances • Housing conditions • Lack of information • Food inaccessibility • Reliance on public transport • Poor mental health
	**Social**	• Lack of social support • Culture shock • Racism
	**Attitudinal**	• “Nowhere to go” • Feeling like a burden • Perception of Canada as safe
**Capacities**	**Material**	• Financial support • Knowledge about pandemic • Mental health supports
	**Social**	• Local social support • Multilingualism
	**Attitudinal**	• Resilience • Religious and spiritual beliefs • “It’s not just about you” • Reflexivity

### 3.1 Material vulnerabilities

International students described six material vulnerabilities that worsened their ability to cope during the first phase of the COVID-19 pandemic: *balancing finances*, *housing conditions*, *lack of information*, *food inaccessibility*, *reliance on public transport*, and *poor mental health*.

#### 3.1.1 Balancing finances

All international students felt their expenses increased and their sources of income decreased during the pandemic, which exacerbated financial strain. Tuition, food, and housing were discussed as the biggest financial burdens. Many international students argued they already pay much more tuition than domestic students, and it was unreasonable for their tuition to increase amid a pandemic, especially as the University had switched to online learning.

Unable to travel back home incurred unexpected costs of living. International students in this study mostly relied on either campus dining plans or takeout for meals, which are both expensive services. Having to extend their use of these services into the summer months, months in which students would otherwise have travelled home, became nearly unbearable. A few students recalled having to choose between paying for rent or food.

Most students discussed that they had jobs prior to the pandemic, but almost half were laid off because of lockdown measures. Those who were still working explained that the Canadian work hour allowance for residents with study permits has never been enough to fund their living and study expenses. International students’ non-permanent resident status also barred them from receiving government benefits received by domestic students, such as the CESB and CERB. Almost all students stated they relied on their parents financially, even though their parents were experiencing financial difficulties themselves because of the pandemic.

*“A lot of students lost their jobs. So, it was definitely a burden on [parents], and even for [those who didn’t], like, although our parents are sending us money from home to study, because of the pandemic, their jobs and everything got affected too. If we were given student grants or loans, it would have been easier on our parents. International students don’t qualify for the [CESB], which is kind of unfair, because we’re still paying for everything. If anything, we’re paying, like four times the tuition [as domestic students]. And it’s not like, they’re not laying off international students either, everything else is the same except for this grant–which in my opinion is really unfair.” (P1).*

#### 3.1.2 Housing conditions

Almost all international students in this study lived with roommates. Sharing often crowded common spaces decreased privacy and increased risk of COVID-19 exposure and transmission. Students emphasized that it was difficult to reduce their risk of COVID-19 because even if they took the necessary precautions, their health was dependent on the decisions and health behaviours of their roommates.

*“As much as you want to social distance, and you know, you want to take all the precautions and avoid as many people as possible, um, with your roommates going out and coming back into the same house, there are moments where you feel like, ‘what if this person just brought in COVID with him, and now like, what if all of us are going to be infected from it?’ You can’t exactly control how safe someone else that is living with you is being.” (P4)*

#### 3.1.3 Lack of information

Nearly all international students expressed they did not have a source for consistent information, especially regarding travel restrictions, about their country of origin. On-campus housing services failed to provide students living on campus with information about COVID-19 cases in their specific buildings, and about health measures they could take to keep themselves safe while living on campus. Information about Canada’s structures and systems, such as the Alberta Residential Tenancy Agreement, also was described as available but “unnavigable” (P3). Difficulties with accessing such important information, in addition to the pandemic context, created taxing situations.

*“[My landlord] did something that she shouldn’t do, by being pretty rude. She knows me and my roommates, like, she thinks we’re international students and we don’t know the Canadian law. And my parents, because they don’t live here, they don’t know how to help. So, like we all moved out. We were looking for houses during the pandemic! It’s been very hard for us.” (P10)*

Many international students felt “bound” (P2) to multiple entities, i.e., people, countries, and organizations. This required them to scour through multiple sources of information, all providing constant and different updates related to these various locales. As a result, students reported feeling distressed and overloaded with information, most of which was unreliable or irrelevant to their situation.

*“It was me trying to keep up with everything that was happening, both in Canada, because I needed to know the rules that I was attached to. And then in my own country, because I still… it was overwhelming. I think a lot of that had to do with the fact that I wasn’t getting the answers I wanted.” (P2)*

#### 3.1.4 Food inaccessibility

Many international students also discussed food inaccessibility, due to long travel times to purchase food and dietary restrictions, as a source of stress. Students living on campus noted that there are no grocery stores within walking distance of the campus. Grocery stores became even more inaccessible during the pandemic because of physical and social distancing requirements. A few students tried grocery delivery services but found they had limited options and long wait times. Students with dietary restrictions, such as eating Halal, were further limited in their food options. A few students described their dependence on a woman living nearby, who provided them with traditional homecooked meals, as their main source of food. However, the pandemic made it difficult for these international students to adhere to their dietary restrictions and continue relying on limited sources of food.

*“We have this Aunty who used to provide us [homecooked] food. That was our main source of food. And when this whole pandemic started, people started to bulk buy, and there was nothing available in the stores. So, she basically gave up on us, she was like ‘I can’t find anything, and you guys are on your own.’ […] We were like, how are we even supposed to eat now?” (P11)*

#### 3.1.5 Reliance on public transport

International students in our study were reliant on public transport for travelling to grocery stores and places of worship. Long travel times in addition to the possibility of crowds increased students’ risk of COVID-19 exposure. Knowing these risks, students were hesitant to access places important to their well-being.

*“It was like my family’s thing to go to [my place of worship] every Sunday. So, after coming here, that obviously reduced a little bit because, you know, I have to take transit and that takes like about an hour and a half to get to the [place of worship]. But ever since the pandemic hit, that reduced a lot more significantly, like I wasn’t able to go to [my place of worship] as often just because I was trying to avoid public transit a lot.” (P4)*

#### 3.1.6 Poor mental health

All of the international students in this study described struggling with poor mental health in daily life as a result of having to juggle different combinations of physical stressors, like academics and finances, with mental stressors, like personal relationships. As a result of the added stress of the pandemic, some students began to engage in unhealthy coping strategies, such as overeating, oversleeping, and overuse of social media, which further exacerbated their mental health.

*“I took food as a stress reliever. I kind of like, from all the stress of not being near my family, from finals, all these outside and inside things piling up, I kind of just tried to solve the anxiety and stress with overeating and like making myself feel calm by just eating.” (P6)*

### 3.2 Social vulnerabilities

International students described three social vulnerabilities: *lack of social support*, *culture shock*, and *racial discrimination*.

#### 3.2.1 Lack of social support

International students explained that they were more socially disadvantaged by the pandemic than domestic students because unlike many domestic students, they were not able to live or spend time with their families. While many international students were able to connect with peers and friends with whom they shared culture, ethnicity, or language, the lack of family created a significant social gap. International students felt that public health measures, such as restrictions on social gatherings, were more detrimental to their social well-being as they overlooked their isolation relative to their domestic counterparts. Students who were unable to find a community of people who shared their culture, ethnicity, or language, were even more susceptible to feeling socially unsupported.

*“I don’t know that many people that speak my language here, because we have so many [languages in my country of origin]. So, it’s like, there’s not like a broad pool of people that you would be able to just like, find people that speak your own language and talk to them and make friends.” (P7)*

#### 3.2.2 Culture shock

First year international students expressed a greater culture shock than those in upper years. However, although upper year students had overcome most of their initial culture shock, they described feeling a novel shock due to Canada’s individualist response to the pandemic. The Canadian government’s hesitancy to take certain public health actions, such as country-wide lockdowns and mandatory mask wearing, surprised many of the students. Students were also frightened by the prevalence of anti-mask protests and some Canadians’ acceptance of conspiracy theories which led to a refusal of following public health guidelines.

*“I know that back home things would have been handled different. Maybe not better, but in a way that as a person who already lived there, you’re already used to it. Here there were all these people who are throwing at me, like their thoughts in this culture. And me coming from another background, I was just like, ‘is that the way I’m supposed to do? Is this what is like, is this what is right now happening?’ […] Here I found so many people trying to tell me don’t wear a mask, and I’m like, it’s a mask, it’s not going to do anything bad to you, at least care about other people […] We back home, we don’t see that the news is against us.” (P2)*

#### 3.2.3 Racism

A few international students described that their identity as a visible minority worsened their experience during the pandemic. Students explained that searching for and retaining employment as a visible minority can be difficult and felt that racism was a determining factor in hiring and termination. Pandemic-related employment scarcity and intermittent business shutdowns made navigating employment as a visible minority even more exasperating.

*“I feel like to a certain extent, there is some racial discrimination here like, behind the scenes, although people aren’t explicit about it. Like job search has been even more difficult because of the pandemic and I feel like me being a visible minority even adds on to that.” (P1)*

Two students described their experience, and hearing about others’ experiences, with Sinophobia.

*“I heard that some people in Canada hate the Chinese because they think this virus*, *it is from China*. *Some people think that it’s*, *um*, *it’s introduced from China on purpose*.*” (P9)*

### 3.3 Attitudinal vulnerabilities

International students explained three attitudinal vulnerabilities: *“nowhere to go”*, *feeling like a burden*, and *perception of Canada as safe*.

#### 3.3.1 “Nowhere to go”

During the pandemic, international students’ “safety mat” (P2), the option of going home in the face of hardship, was taken away, leaving students feeling like they had “nowhere to go” (P5). Exclusion from government supports worsened feelings of isolation and abandonment. Nearly all students expressed internalizing this abandonment, and consequently, felt a pessimistic inclination to be wholly self-reliant in the future.

*“No one really talked about what it meant for us, going online, as international students. One thing that was completely eye-opening was when I finally came to the conclusion that as an international student, your last resort is, ‘if something happens, I’m going home.’ What happens when your last resort, the one you always have as a safety mat, kind of like disappears out of the blue? And that is something that I will tell my friends […] I learned that I should not just rely on my safety mat. Everything about like [the pandemic], I realized I’m the only one that can and is helping myself.” (P2)*

#### 3.3.2 Feeling like a burden

Nearly all students relied on their parents as a source of income. Despite trying to live frugally, international students harboured feelings of guilt; studying abroad is expensive and the exchange rate of their country of origin’s currency to Canadian dollars can be weak. As the pandemic distressed economies globally, students felt even more guilty relying on their parents for financial support.

*“I’ve kind of always felt like a burden that, you know, it’s so expensive, like studying abroad. I’ve always wanted to like, my partner, for example, he works a lot […] and he has a very frugal lifestyle […] I really wish I could be on that level, so I could slowly take that burden off my dad. That’s something I’ve always felt, like… The older I got, I was like, I should really start, you know, finding ways to help my dad even more. But yeah, in the pandemic for sure. I felt like that need to help my dad out increase for sure.” (P3)*

Some students added that their families were constantly worried about their health and safety, concerns which only swelled during the pandemic. Students felt that these concerns were a burden on their family; if they were at home, their families would not have to worry as much.

#### 3.3.3 Perception of Canada as safe

Roughly half of the international students described perceiving Canada as a safe place to live and study. As a result, a few students felt less susceptible to COVID-19, and participated in behaviours, such as frequently eating out, which could have increased their risk of infection.

*“We live in a country where the government and health systems take care of precautions properly […] you have the courage to go out and do a lot of things versus if you lived in a country where you know, proper safety wasn’t ensured. I know in [my country of origin], most people don’t go out as much because it’s not safe […] So, I still eat like, five times outside per week because obviously it’s a good way of meeting people. Every time I’m just like, ‘oh, I haven’t met someone in a while’, I make a plan to eat outside.” (P1)*

### 3.4 Material capacities

International students discussed three material capacities that strengthened their ability to cope during the first phase of the COVID-19 pandemic: *financial support*, *knowledge about pandemic*, and *mental health supports*.

#### 3.4.1 Financial support

The majority of students expressed a sense of security in knowing they had financial support from their parents. Because of this support, they would have their necessities covered, even if they were unable to find a job in Canada. Some embassies and local ethnic organizations also provided funds and other material supports during the pandemic.

*“[My country of origin’s] embassy, did, in like June, gift cards of $200–300 of food. I know that [another] embassy they brought a package of like supplies, like personal hygiene supplies and things like that for their students and also like some food. Many embassies did the same approach as mine where they were just doing the money kind of support. And I have a friend who, they were offering her what they call a ‘humanitarian flight’.” (P2)*

#### 3.4.2 Knowledge about pandemic

All students had knowledge about the public health measures necessary to reducing their risk of COVID-19, and of the government supports and restrictions introduced because of the pandemic. Most students stated they encountered this information on social media, group chats, and University emails–even if they were not actively searching for it.

*“[My ethnic group chats] always send information, like, how to protect yourself. I get a lot of information like that. I felt better prepared that when I get out of my room, I will take this precaution and that precaution.” (P9)*

#### 3.4.3 Mental health supports

During the pandemic, students felt that communication with their families over the phone was an unwavering means of emotional support because they were accustomed to communicating with their families over the phone regularly before the pandemic anyway.

*“My entire family is back home in [country of origin], so family support wasn’t really affected, because it was all over the phone anyway. I would say that was still the one constant throughout the whole thing, that I was still talking to them over the phone and stuff.” (P7)*

Many students appreciated that the University’s mental health facilities and alternative grading options were accessible and supportive for their mental wellbeing.

*“The school sent emails about wellness, mental health or something, and they were like, ‘oh, if you need help contact us.’ […] Also, in class online, one of my professors was like, ‘if you guys feel stressed out and scared or something, feel free to, like, talk to me or the school, the [on-campus health and wellness centre]’. So that made me feel like supported […] It let me feel like someone’s caring about me besides just me and my family. It doesn’t matter if I want to go or not, but it makes me feel like something is there for help.” (P10)*

### 3.5 Social capacities

International students discussed two social capacities: *local social support* and *multilingualism*.

#### 3.5.1 Local social support

Students described that their existing social networks in Canada supported them greatly during the pandemic. A few students had relatives nearby who they were able to visit or live with, which offered a sense of family support and helped offset some of the unexpected expenses students faced because of the pandemic. As well, students explained that living with roommates greatly cultivated their emotional and social wellbeing. Roommates tended to be ethnically similar international students and therefore were all experiencing similar hardships. If not already a close group of friends, students explained that because they were all in the same situation, they naturally became close friends.

*“I think it helped me out a lot, mental health-wise, because like when you’re living with friends, it’s like a second family. That was a home for me at that particular time. All of us were international students and we were all from [the same country of origin] […] It made our quarantine better by staying together since we were all stranded in the same situation anyway.” (P11)*

A few international students felt supported by their local religious communities. One student noted the community offered material support to Muslim international students during the month of Ramadan. Another student had opportunities to talk to others on a religious basis.

*“The [place of worship] I attend they do a program for teenagers or like university students. We’d just talk about random stuff and share the [religious scripture] a bit. So yeah, I’d say it made my health better. I’ll say it’s helped me like, feel like there are people to talk to about these kinds of things.” (P8)*

#### 3.5.2 Multilingualism

Nearly all students stated that their ability to speak more than one language enabled them to access diverse sources of information and build more meaningful friendships.

*“I feel that in general knowing more languages, it helps you to understand the world better and in a more complex way. I understand English pretty well, it helped me because I can read like the news and maybe like more science related news, than people that do not understand English as well. And also, what helped me a lot is that my brother studies medicine […] he explained everything to me in [my native language] […] I feel that it gave me an advantage because I had different sources of information.” (P5)*

Some students felt more comfortable engaging with students with whom they shared languages and were also inclined to develop more meaningful relationships with these students. In particular, international students who were in their first year when the University switched to online learning found that students who shared their language were more likely to also share ethnicities, cultures, and experiences. This helped buffer the effects of culture shock and improve collective mental and social wellbeing.

*“Once I found that there were like, more international students who also [spoke my language] in my building, I was very happy because like, although we can come from different countries, there are some similar experiences. So, we could like, bond over that, like music, language, culture and things like that. They were just like, an additional source of support that would understand my situation more than my Canadian friends. My friends gave me emotional support as well, and because they were going through like the same situation as me, it was like, I felt like um, I didn’t feel alone. (P5)*

### 3.6 Attitudinal capacities

International students discussed four attitudinal capacities: *resilience*, *religious and spiritual beliefs*, *“it’s not just about you”*, and *reflexivity*.

#### 3.6.1 Resilience

All students explained that they undergo many “big changes” (P5) when studying abroad; for the first time they are away from their families, independent, and encounter culture shock. Many students expressed that their experience adapting to and overcoming these changes enabled them to overcome pandemic-related challenges with less apprehension than others.

*“I’ve lived my whole life in [my country of origin]. So, it was a big change for me going to Canada, and that big change like helped me to adapt to situations like the pandemic more easily. And not only like moving to Canada, but just like, experiencing the whole, like Canadian university life. It has kind of mentally prepared me to be able to adapt more easily to change.” (P5)*

Three female international students described feeling an added layer of confidence in their resilience because they had to worry less about certain necessities, such as safety and security, in Canada than in their country of origin. These students felt empowered believing they could be stronger, more independent, and more resilient in Canada.

*“Feeling secure as a woman in [my country of origin] is way different than a woman in Calgary. Like I would personally… I’ve never, like whenever I used to go out, I would never feel fully safe or comfortable. You know, like you have lots of people who just stare at you on the streets […] So I would find it difficult to go out by myself. That’s much less here.” (P3)*

#### 3.6.2 Religious and spiritual beliefs

Roughly half of the international students were religious or spiritual. These students explained their religious or spiritual beliefs had served as a constant positive coping mechanism throughout their life. For instance, a few students relied on religion as an internal constant when, upon migrating, their external cultural environment changed drastically. Accordingly, students’ religious or spiritual beliefs helped strengthen their mental health during the pandemic, and also encouraged them to take the necessary precautions to sustain their physical health.

*“I think something that made my transition to Canada easier was just me basing my values on my religion rather than culture […] as long as I’m following my religion, and as long as I’m not hurting anyone, I don’t really care about what’s different culturally […] I also believe a saying, ‘I, God, am what my servant thinks of me’, which means that whatever I think of God or whatever I think God will do for me will occur. So, I kept thinking that God won’t allow anything to hurt me as long as I take all the precautions I can. In our religion, it’s important to like both believe and work for it. That too added to why I wasn’t bothered or scared as much [during the pandemic].” (P6)*

#### 3.6.3 “It’s not just about you”

All international students expressed shock at some Canadians’ refusal to follow public health guidelines during the pandemic. Coming from predominantly collectivist societies, students inherently possessed a cooperative mentality which prioritizes the common good over individual liberties, and places trust in governments to act in favour of the collective. Nearly all students therefore were better equipped to adapt to disruptions to their personal lives, emphasizing that “it’s not just about you” (P7). Students’ readiness to adhere to imposed public health guidelines reduced their own risk of COVID-19 infection and the risk of transmission to those around them.

*“For us, our government telling us wear a mask, it’s not something that we’re going to say no to […] We actually learn to just accept the fact that like, you not wearing a mask puts someone in danger […] That was where I really believe my background as an ethnic person comes in, or at least in the type of like group that I was brought up in, wearing a mask is not a big deal.” (P2)*

#### 3.6.4 Reflexivity

All students described reflexivity–a tendency to be self-aware and reflect on their strengths and weaknesses, both intrinsically and extrinsically. This reflexivity enabled students to recognize and be more grateful for their privileges on a daily basis. In turn, students explained they were able to be more resourceful in shaping even the most dreadful circumstances, such as the global standstill caused by the pandemic, to be advantageous for them, which benefited their mental wellbeing. All students took up hobbies, learned new skills, set aside time for self-care, or found ways to benefit from online events and schooling.

*“I had time to self-reflect and appreciate more things in life […] So, I just basically, like, fixed my mental health and did things that I otherwise wouldn’t do […] When the pandemic hit and school went online, I was just like I definitely can’t take [my negative] kind of attitude into the next semester, so I had to think of ways to buckle up for like the upcoming semesters. Then I realized that okay, since it’s online, I can use it to my advantage. […] During in-person school I couldn’t sleep until like 3am, and I had 8am classes. So, fixing my sleep schedule has helped me a lot. […] Normally, going to [my place of worship] I’d have to wake up, take the bus, come back, sometimes if I’m lazy I’d use Uber to get there on time. But when the pandemic hit and I started online [worship], like, that was a sweet blessing because I didn’t have to go anywhere, and I’ll say it made my spiritual life much better.” (P8)*

## 4. Discussion

This study used the CVA framework to identify the vulnerabilities and capacities of international students studying at a university in Calgary, Canada during the first phase of the COVID-19 pandemic. Our findings provide important insights into the overlapping and complex relationships between students’ vulnerabilities and capacities, and the ways in which students’ capacities may be leveraged to help them cope and recover from disasters.

Many of the factors we identified as vulnerabilities are consistent with findings from previous literature, such as financial strain [28], work hour restrictions [29], travel restrictions [30], exclusion from government supports [31], crowded housing [20], inaccessible information [19], feelings of isolation [30, 32], discrimination [30, 33], and culture shock [9].

However, we found that factors categorically labelled as vulnerabilities by previous literature were complex, and in some cases were leveraged by international students as capacities to offset their vulnerabilities. Living in crowded housing increased students’ risk of COVID-19, reduced privacy, and affected their ability to study. Simultaneously, living with roommates cultivated a supportive social network that alleviated international students’ feelings of isolation. Those sharing a dietary restriction specifically described feeling united in dealing with reductions in food options catering to their restriction during the pandemic. Social support was similarly regarded as a salient factor in helping students mitigate pandemic-related hurdles among international students in the United States [30].

Language difficulties are described by established literature as the most prevalent vulnerability for international students [9], but students in our study explained that their multilingualism provided opportunities to build meaningful social networks and access diverse sources of information during the pandemic.

Nearly all students in our study agreed that their experience moving away from their families to study abroad was difficult, but it uniquely advantaged them in having the resilience to adapt to the pandemic. A similar outcome was identified among Latino migrants; because they experienced more stressors in daily life, migrants were uniquely able to cope with the H1N1 pandemic with less apprehension than non-migrants [34]. Interestingly, resilience appeared to be more pronounced among some female students, a finding that has been supported by some quantitative research previously [35] but warrants further investigation. Female international students studying in the United States and Canada have been found to adjust to their host country better and faster than males because these countries typically afford greater gender equality than their countries of origin [36].

Culture shock is another factor commonly perceived as a vulnerability. However, predominantly coming from countries with collectivist societies, students described that their background better equipped them to adhere to public health guidelines during the pandemic as collectivism prioritizes the common good over individual liberties. Ethnic minorities were more likely to adopt preventative behaviours during the H1N1 pandemic, possibly due to similar reasons [37].

International students’ religious and spiritual beliefs also served as constant positive coping mechanisms during the pandemic. Similarly, international students with a religious affiliation showed higher levels of resilience than students without in the United States [38]. Some students in our study explained that there was no point in being anxious about contracting COVID-19, because as long as they took all necessary precautions, they could leave the rest to God. A similar belief held by Latino migrants, known as *destino*, fostered their resilience during the H1N1 pandemic [34].

These findings highlight international students’ resourcefulness and resilience and suggest that there is a more complex relationship between vulnerabilities and capacities than previously described; factors in daily life that may weaken students’ ability to cope with disaster could also be leveraged to strengthen students’ ability to recover from disaster. Emerging research has identified “silver linings” of the pandemic among other populations as well [39]. European studies of adults in the general population found that the opportunity to reflect, grow, and take up enjoyable activities were prominent positive experiences during the pandemic, similar to our theme of reflexivity [39–41]. There is a need to re-examine vulnerability-focused narratives in disaster-related literature, especially the idea that non-Western values are barriers to success in Western societies [11].

### 4.1 Implications

Our findings have implications for research, policy, and practice related to the international student experience, wellbeing, and academic success. The most prominent vulnerability in our study, similar to established literature, was financial burden. To alleviate this burden, the Canadian government can include international students in future emergency benefits and increase their work hour allowance [31]. Students’ countries of origin could also capitalize on embassies as a point of contact to support students.

Universities have a responsibility and “privileged position” to serve as a reliable and local information source for students during disasters [18]. University departments can aim to coordinate and disseminate accessible, personalized, and comprehensible information in times of crisis for its international student population. Universities can also promote international students’ existing capacities, such as resourcefulness and cultural, religious, and spiritual beliefs, as well as facilitate local social networks, through culturally competent programming.

### 4.2 Strengths and limitations

Our use of the CVA framework to facilitate a consistent structure throughout the data collection and interpretation process was a key strength of this study, as it elucidated that international students have many capacities that overlap with and can offset their vulnerabilities.

The findings from our study may need to be interpreted with caution because of our small sample size. Our recruitment involved convenience sampling and snowball sampling, which can overrepresent those participants and their social networks who volunteer, or the researcher contacts, first [42]. Existing literature reports a lack of generalizability as a possible limitation of convenience sampling. However, given the nature of qualitative inquiry, our study did not aim for generalizability. We instead took steps to support transferability, such as by reporting thick descriptions of our participants. On the other hand, snowball sampling can be useful for locating harder-to-reach populations [43]. Many international students had travelled back home, and public health guidelines restricted our study’s advertising to online means–both factors which made our study population more difficult to reach.

We were unable to aim for meaning saturation in our study, which typically requires 16–24 interviews [26]. Nonetheless, this study included more than the nine interviews typically necessary for achieving code saturation, and aiming for code saturation was sufficient for our study objective.

We also acknowledge the potential role of researcher positionality in this study [44]. The primary investigator is a woman, Muslim, South Asian, first-generation immigrant, and was a domestic undergraduate University of Calgary student at the time this study was conducted. The experiences of the primary investigator during the COVID-19 pandemic may overlap with some of the experiences of international students who associate with similar identities. The primary investigator took an insider position in relation to the participants when engaging with these identities in order to access a more meaningful and authentic understanding of students’ experiences. As well, we were able to engage all participants in member checking of our results, which further supported the credibility of our study.

## 5. Conclusions

The purpose of this study was to explore the vulnerabilities and capacities of international students studying at a university in Calgary, Canada during the first phase of the COVID-19 pandemic. We found complex relationships indicating that while international students’ vulnerabilities were exacerbated and introduced challenges during the pandemic, students uniquely leveraged their capacities which strengthened their ability to cope with and recover from challenges. Findings from this study may be informative for stakeholders involved in disaster response, such as universities, health professionals, and governmental and non-governmental institutions, to shift focus towards cultivating international students’ capacities to ensure vulnerabilities are sustainably alleviated. Actionable steps may include international students’ inclusion in emergency benefits, using embassies as a point of contact, disseminating accessible and relevant information, and culturally competent programming to build capacities.

## Supporting information

S1 File Interview guide. (DOCX)
