# Supplementary material for: “It’s not just about you”: International students’ vulnerabilities and capacities during the first phase of the COVID-19 pandemic in Canada
Source: PLoS One. 2024 Oct 3;19(10):e0311514. doi: 10.1371/journal.pone.0311514 (PMC11449335; doi:10.1371/journal.pone.0311514)
Supplement: S1 File — (DOCX) [file pone.0311514.s001.docx]

**Interview Guide**

**Introduction and Consent**

*Introduce researchers present.*

*Go over study information from the Informed Oral Consent Document.* Thank you for agreeing to participate in this study about the vulnerabilities and capacities of Canadian postsecondary international students during the COVID-19 pandemic. I hope you had the chance to look over the Informed Oral Consent Document I sent to you yesterday – did you have any questions about the study or the Document?

I will summarize the key points from the Informed Oral Consent Document again: We will be asking you questions about your experiences during the COVID-19 pandemic. With your oral consent, we will be audio-recording your responses, so please speak clearly and note that the recorder will not pick up actions such as nodding in agreement. All of your responses will be kept confidential. There are no right or wrong answers to any question. There are no risks to participating in this study. If at any point you wish to skip a question or stop the interview entirely, that is completely up to you.

*Start audio-recording with permission.*

*Oral consent.* Your decision of allowing the researcher to ask you questions over Zoom will be interpreted as an indication of your agreement to participate. In no way does this waive your legal rights nor release the investigators or involved institutions from their legal and professional responsibilities. You are free to withdraw from the study at any time. Do you agree to take part in the study?

**Demographic Questions**

1. What is your age?
2. What is your current year of study?
3. What gender do you identify with?
4. What is your employment status? (FT/PT/Unemployed)
5. What languages do you speak fluently?
6. How would you describe your ethnicity?
7. What is your country of origin?
8. How many years have you lived in Canada? (< 1 year, 1 year, > 1 year)
9. What was your type of residence when the pandemic hit, so in March 2020?
   - On-campus individually
   - On-campus with roommates
   - On-campus with family
   - Off-campus individually
   - Off-campus with roommates
   - Off-campus with family
   - Other, please specify: ______

**Open-Ended Questions**

*The COVID-19 pandemic has been difficult for everyone. What have been standout moments for you, either positive or negative, during the pandemic?*

**Material**

1. Do you feel your physical or mental health affected your ability to *cope* *with and recover from* the COVID-19 pandemic and/or associated public health guidelines (e.g., face masks, physical distancing, quarantine)? How so?
   - Do you feel the COVID-19 pandemic affected your physical or mental health? How so?
   - Did you ever feel your physical or mental health affected your daily life before the pandemic? How so?
2. Do you feel your housing situation/type of residence has affected your ability to *cope* *with and recover from* the COVID-19 pandemic? How so?
   - Do you feel the COVID-19 pandemic affected your housing situation? How so?
   - Did you ever feel your housing situation affected your daily life before the pandemic? How so?
3. Do you feel your financial situation/income affected your ability to *cope* *with and recover from* the COVID-19 pandemic? How so?
   - Do you feel the COVID-19 pandemic affected your financial situation/income?
   - Did you ever feel your financial situation/income affected your daily life before the pandemic? How so?
4. Do you feel your level of food security affected your ability to *cope* *with and recover from* the COVID-19 pandemic? How so?
   - Do you feel the COVID-19 pandemic affected your food security? How?
   - Did you ever feel your food security affected your daily life before the pandemic?

**Social**

1. Do you feel your status as an international student (as distinct from domestic students) has affected your ability to *cope* *with and recover from* the COVID-19 pandemic? How?
   - Did you ever feel your status as an international student affected your daily life before the pandemic? How so?
2. Do you feel your social networks (e.g., family, friends, communities) have affected your ability to *cope* *with and recover from* the COVID-19 pandemic? How so?
   - Do you feel the COVID-19 pandemic has affected your social networks? How so?
   - How did your social networks affect your daily life before the pandemic?
3. Do you feel your diverse language skills have affected your ability to *cope* *with and recover from* the COVID-19 pandemic? How so?
   - Did you ever feel your diverse language skills affected your daily life before the pandemic? How so?
4. Do you feel your race or ethnicity has affected your ability to *cope* *with and recover from* the COVID-19 pandemic? How so?
   - Did you ever feel your race or ethnicity affected your daily life before the pandemic?
5. Do you feel your gender has affected your ability to *cope* *with and recover from* the COVID-19 pandemic? How so?
   - Did you ever feel your gender affected your daily life before the pandemic?
6. Do you feel your religious/spiritual or cultural beliefs have affected your ability to *cope* *with and recover from* the COVID-19 pandemic? How so?
   - Did you ever feel your religious or cultural beliefs affected your daily life before the pandemic? How so?

**Attitudinal**

1. How did you feel during the first few months of the pandemic?
   - Does this differ from how you feel now?
2. Have you felt prepared to *cope* *with and recover from* the COVID-19 pandemic and/or associated public health guidelines? Why or why not?
   - Do you feel prepared to tackle the future after the pandemic? Why or why not?
   - Did you feel prepared to tackle daily life before the pandemic? Why or why not?
3. Have you felt supported (e.g., by friends/family, university, government, organizations) to *cope* *with and recover from* the COVID-19 pandemic and/or associated public health guidelines? Why or why not?
   - Do you feel supported to tackle the future after the pandemic? Why or why not?
   - Did you feel supported to tackle daily life before the pandemic? Why or why not?
4. Have you felt safe to *deal* with the COVID-19 pandemic? Why or why not?
   - Do you feel safe to cope with the future after the pandemic? Why or why not?
   - Did you feel safe to cope with daily life before the pandemic? Why or why not?
5. Have you felt the COVID-19 pandemic and associated public health guidelines panned out according to your hopes and expectations? Why or why not?
   - Do you feel your future will pan out according to your hopes and expectations after the pandemic? Why or why not?
   - Did you feel your daily life was panning out according to your hopes and expectations before the COVID-19 pandemic? Why or why not?
6. Have you felt optimistic and motivated to *cope* *with and recover from* with the COVID-19 pandemic? Why or why not?
   - Do you feel optimistic and motivated to deal with the future after the pandemic? Why or why not?
   - Did you feel optimistic and motivated to deal with daily life before the COVID-19 pandemic? Why or why not?

*If you had to choose three things that best helped you navigate the pandemic from March till now, what would they be?*

*If you had to choose three things that were the biggest challenges in navigating the pandemic from March till now, what would they be?*

*Conclusion.* That concludes my questions for the interview, thank you for your support. Is there anything else you would like to add?
